# Manuel de Technique des Autopsies

**Published:** 1886-10

**Authors:** 


MANUEL DE TECHNIQUE DES AUTOPSIES. *Par* BOURNEVILLE *et* P. BRICON. *Duodeceimo*, *pp. xii*, *240. Paris.*

This is an interesting work on post-mortem examinations; when and how to make them.

For internes in hospitals, this little volume is an invaluable guide; but in private post-mortem examinations, as well as those performed before a class, it would be impracticable to observe all the details suggested by the authors. In many cases time is too limited to admit it.

In order to profit most by it, post-mortem examination ought to be made as soon after death as possible.

This volume has two objects in view—

1. To attract attention to the importance ot careful observation in autopsies, and to encourage correct teaching of pathological anatomy.

2. To teach students to make better and quicker post-mortem examinations in hospitals.

The work is clear and concise, and deserves to be welcomed by physicians and students, and especially by the pathological anatomist. D. D.

